# A Snap in the Night: A Case Report of Penile Fracture

**DOI:** 10.1002/ccr3.71209

**Published:** 2025-10-08

**Authors:** Dennis Awedam Achio, Richmond Buckner, Mary Monney‐Bortey, Celestine Tsogbe, Bernice Ahiadormey, Priscilla Mary Tetteh, Eunice Wilberforce A. Achio

**Affiliations:** ^1^ Urology Department University of Ghana Medical Center Accra Ghana; ^2^ Department of Family Medicine University of Ghana Medical Center Accra Ghana

**Keywords:** case report, penile fracture, tunica albuginea, urological emergency

## Abstract

Penile fracture is a rare urological emergency that requires prompt diagnosis and treatment. We report a case of a 44‐year‐old male who sustained a penile fracture during sexual intercourse. Surgical exploration and repair were performed within 10 h. The patient experienced an uneventful recovery, highlighting the importance of early surgical management.

## 1 Introduction

Penile fracture is a rare but serious urological emergency characterized by the rupture of the tunica albuginea, the fibrous tissue layer enveloping the corpus cavernosum [1].

This injury typically results from blunt trauma to the erect penis, with vigorous sexual activity being the most common cause, followed by other activities such as manual manipulation or rolling over in bed. The tunica albuginea, which becomes significantly thinner and more vulnerable during an erection, cannot withstand the shearing forces imposed by sudden bending or direct impact, leading to its rupture [2]. The clinical presentation of penile fracture is often dramatic. It includes a sudden audible “popping” sound, acute pain, rapid detumescence, and swelling of the penis, frequently accompanied by a characteristic hematoma and deformity, commonly referred to as the “eggplant sign^”^ [3]. In some cases, urethral injury may also occur, manifesting as blood at the meatus or difficulty in urination. Urethral involvement occurs in approximately 10%–38% of cases and warrants careful evaluation, especially if haematuria or urinary retention is present [4].

Prompt diagnosis and early surgical intervention are critical in the management of penile fractures to minimize the risk of long‐term complications, such as erectile dysfunction, penile curvature, painful erections, or psychological distress. Studies have shown that early surgical repair, ideally within 24 h, results in significantly better functional outcomes compared to delayed or conservative treatment approaches [5]. Delayed intervention has been associated with higher rates of fibrosis, chronic pain, and sexual dysfunction [6], emphasizing the importance of timely repair to restore penile anatomy and function.

The article is noteworthy for its clear classic presentation with an audible “snap”, immediate clinical recognition, and prompt surgical repair leading to full restoration or improved ability to achieve and maintain an erection sufficient for sexual intercourse, offering a practical reference for optimal management of similar emergencies.

This article presents a case of penile fracture in a 44‐year‐old male, emphasizing the clinical features, diagnostic approach, and successful surgical management, with a discussion of relevant literature to highlight the importance of early intervention in restoring erection and preventing penile curvature.

## 2 Case History/Examination

We present a 44‐year‐old male who came to the emergency department with a swollen, deformed penis and acute pain following sexual intercourse with his wife an hour prior to presentation. He admitted that during vigorous thrusting and in a “doggy” sex position, his penis accidentally struck her buttocks with significant force, causing an audible “pop” sound, immediate loss of erection, and severe pain. He noticed immediate penile swelling and discoloration.

**FIGURE 1:**
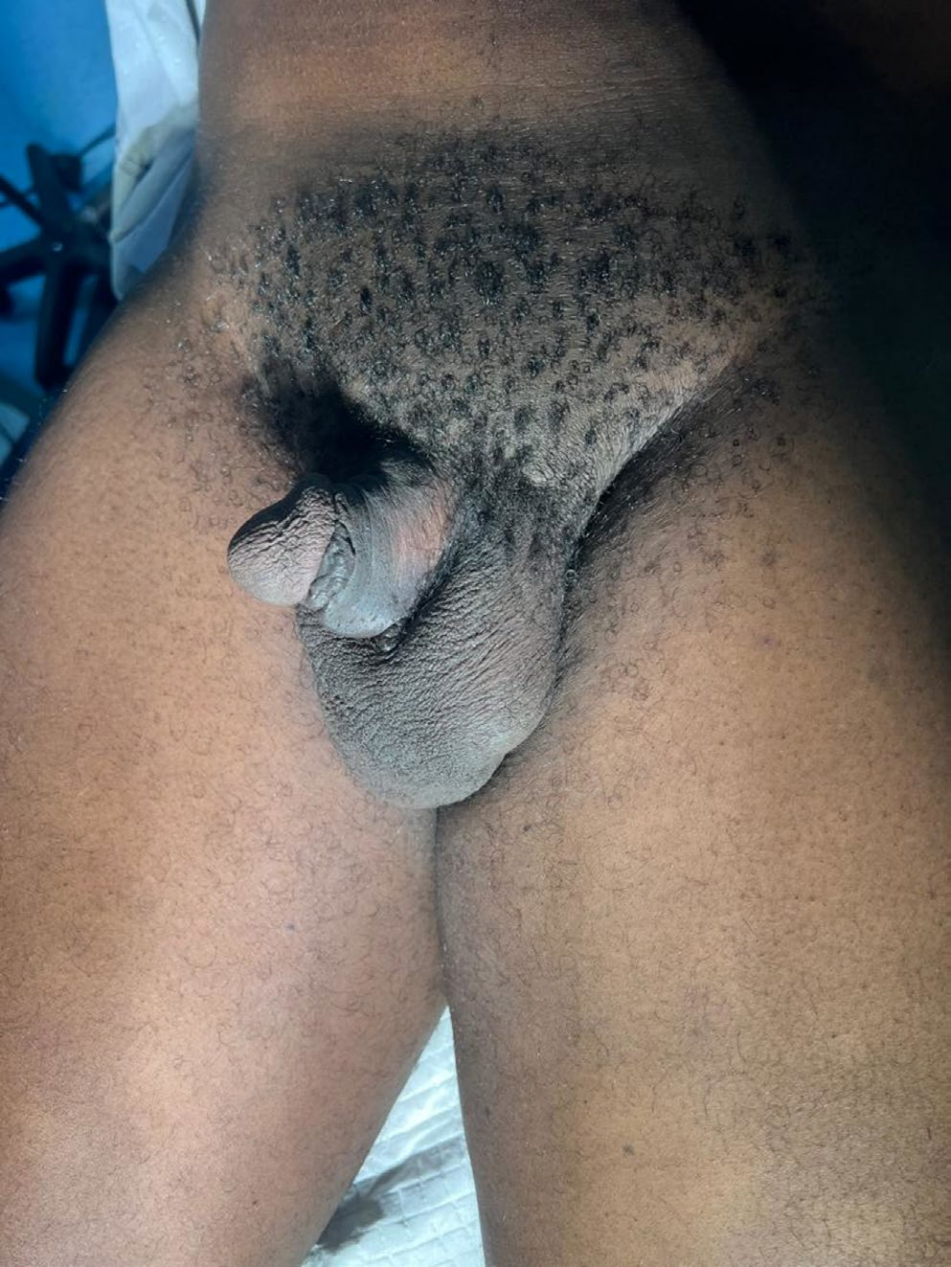
Deformed penile shaft.

On physical examination, as seen in Figure 1, the penis was swollen and deformed. There was abnormal angulation and tenderness over the penile shaft without the involvement of the glans penis. There was no sign of blood at the urethral meatus and no difficulty with voiding.

A diagnosis of penile fracture was made based on clinical findings and a detailed history, including the mechanism of injury and sudden detumescence. An ultrasound was not done given the clear presentation.

Analgesics were administered to help with the pain, and formal consent was sought for surgery. The patient was hydrated with intravenous fluids, and prophylactic antibiotics were given. Full blood count, blood urea, creatinine, and urine routine examination results were unremarkable.

**FIGURE 2:**
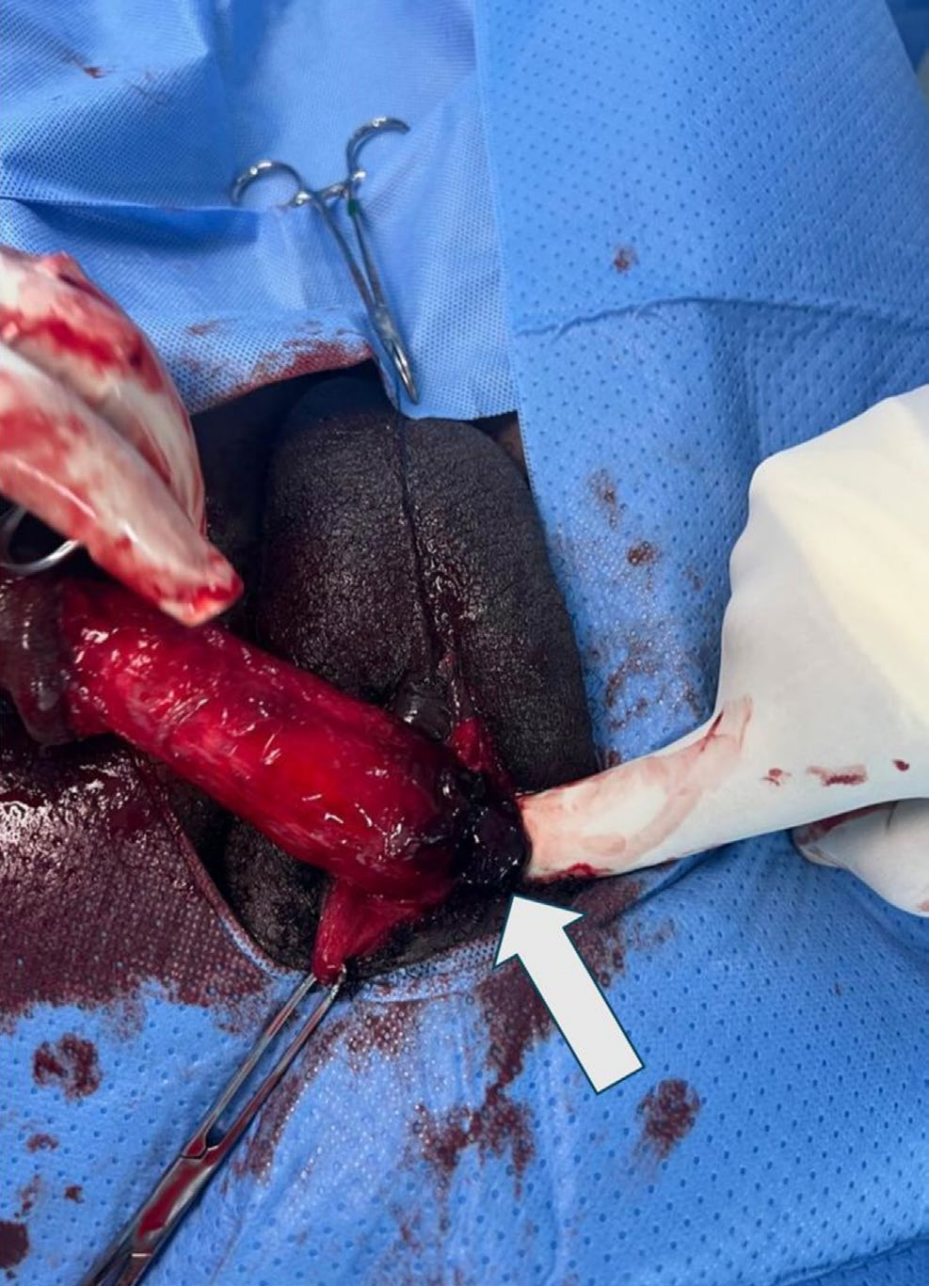
Hematoma at site of tear.

**FIGURE 3:**
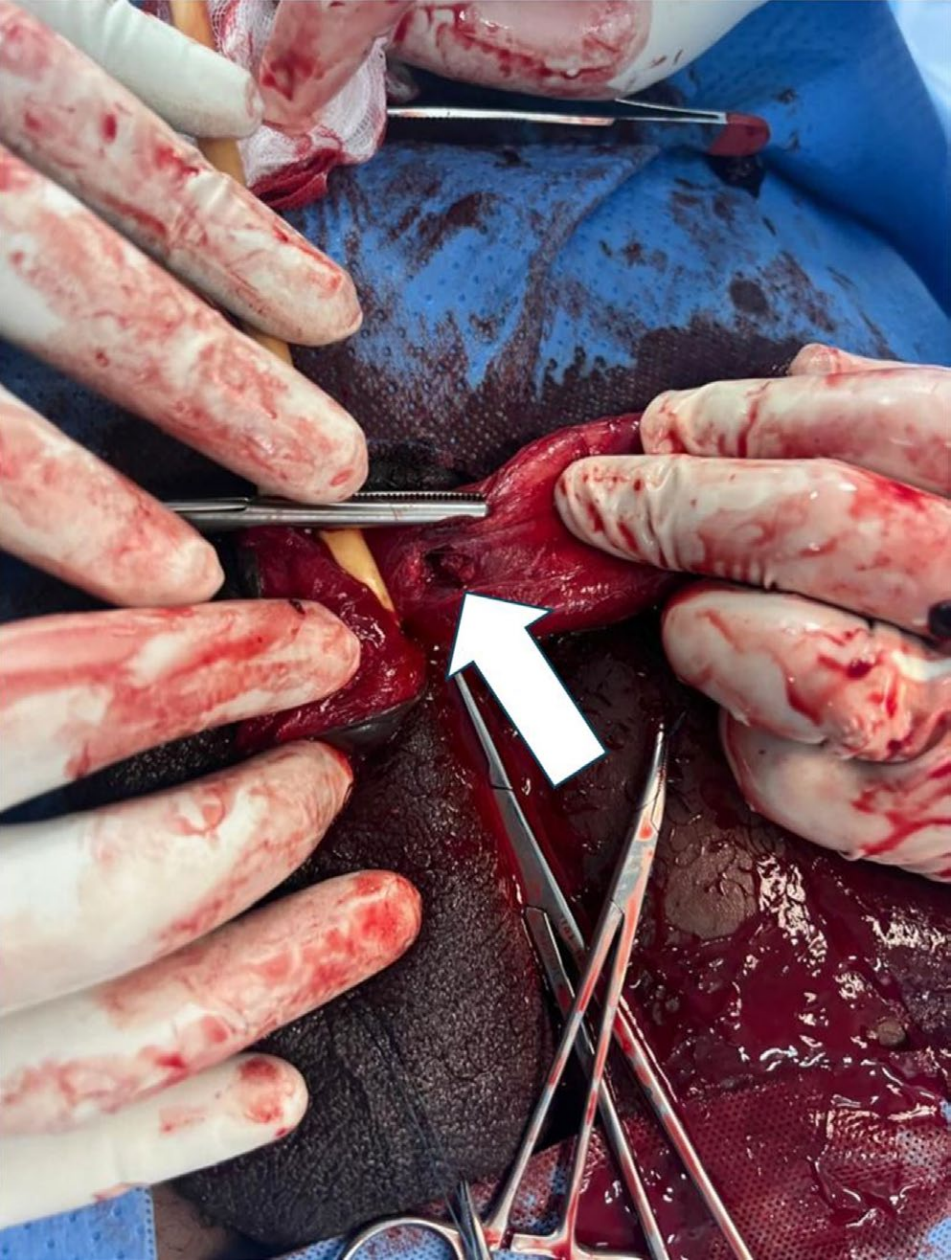
Tear in the tunica albuginea.

**FIGURE 4:**
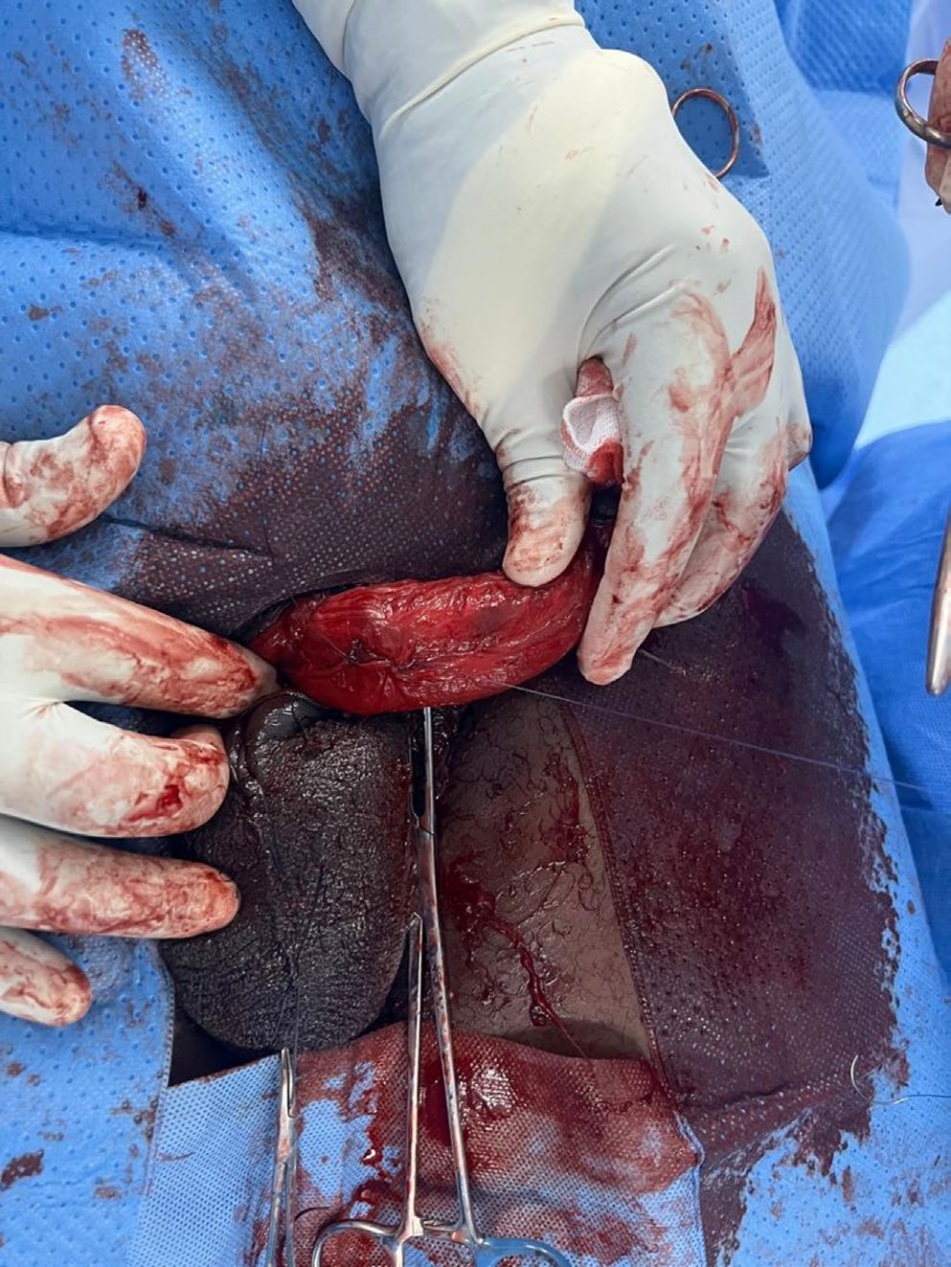
Tunica albuginea repaired.

**FIGURE 5:**
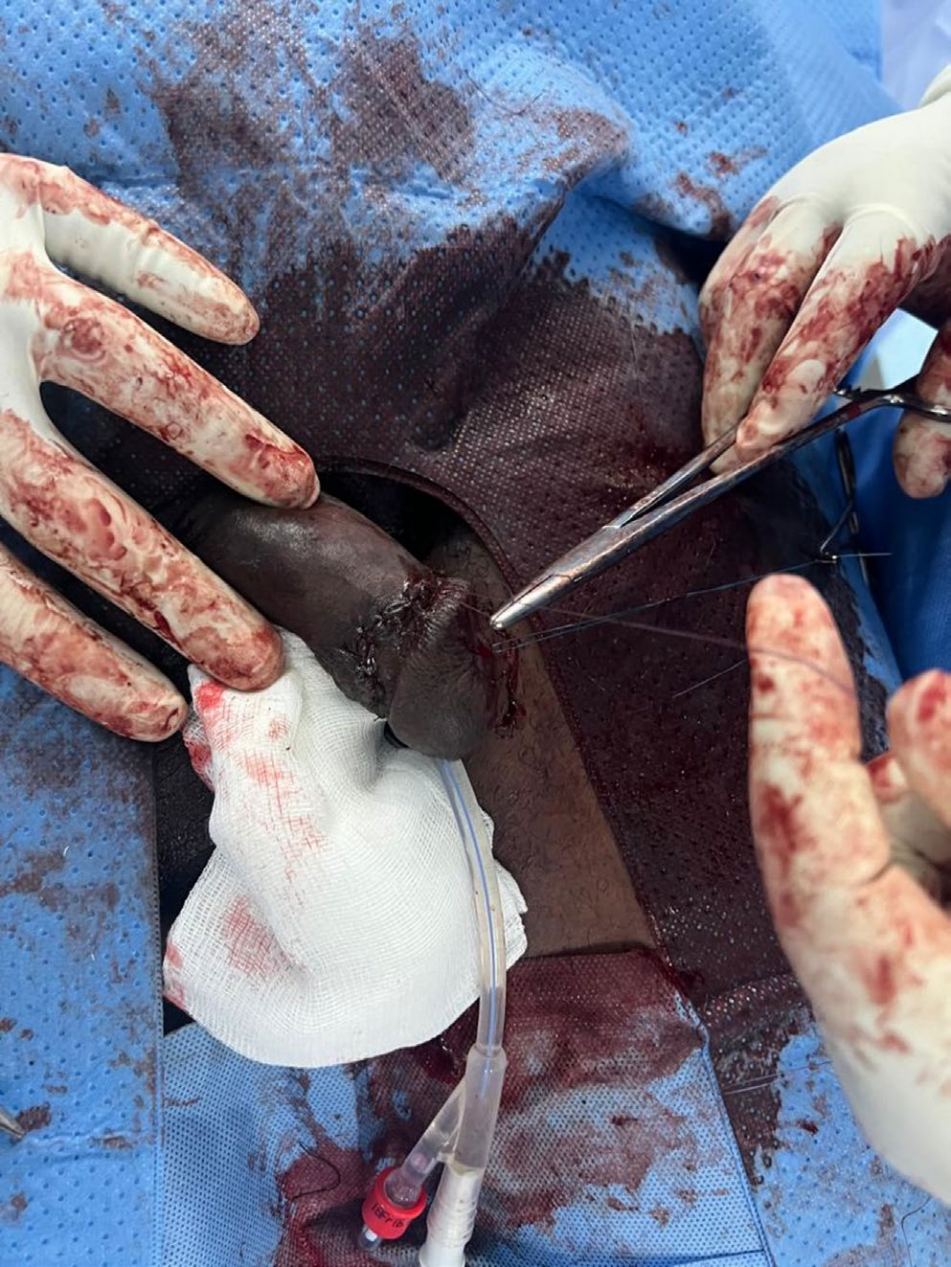
Skin incision closed.

The patient was taken to the operating room within 10 h of injury. A urethral catheter was passed. Under spinal anesthesia, a circumferential degloving incision was made on the skin, being mindful of the urethra. The skin was pulled down. Close to the base of the penis showed dark hematoma which measured about 5 mLs, as seen in Figure 2, and a tear, approximately 1.2 cm in the tunica albuginea of the right corpus cavernosum, as seen in Figure 3. The tear was carefully repaired with absorbable sutures, achieving satisfactory closure, as seen in Figure 4. The skin was pulled up for closure, as seen in Figure 5. On postoperative day two, the urethral catheter was removed; the patient voided freely, and the uroflowmetry test showed a Qmax of 27. The patient was discharged after a thorough examination on antibiotics and analgesics.

All images are published with the patient's written concern.

## 3 Differential Diagnosis

Penile fracture.

Penile hematoma without tunica rupture.

Superficial dorsal vein rupture.

Urethral injury.

## 4 Conclusion and Results

Penile fractures should be managed promptly with surgical exploration and repair. This case underscores the importance of timely intervention to restore the ability to achieve and maintain an erection sufficient for sexual intercourse, preventing long‐term complications in patients presenting with penile fracture. The patient was advised to abstain from sexual activity for 8 weeks post operation and completed follow‐ups at 1, 3, and 6 months postsurgery, with wound dressing being done every other day until the wound is completely healed. From the 3‐month follow‐up, the patient reported satisfactory erectile function. At 6 months, the patient's International Index of Erectile Function (IIEF)‐5 score was 22, and the Uroflowmetry test showed a Qmax of 28, with no pain, penile curvature, or difficulty with urination.

## 5 Discussion

Penile fracture is an uncommon but critical urological emergency that necessitates prompt recognition and intervention. It is characterized by the rupture of the tunica albuginea, which surrounds the corpora cavernosa. This condition is most commonly caused by blunt trauma to the erect penis during sexual activity. However, other mechanisms such as manual manipulation, falls, or rolling over in bed are also reported [7]. The incidence of penile fracture varies globally, influenced by cultural, anatomical, and behavioral factors. Studies from Western regions report that over 50% of penile fractures occur during heterosexual intercourse, often associated with positions like “woman‐on‐top” or “doggy style” that limit male control over thrusting movements [8]. A study by Barros et al. on the relationship between sexual position and the severity of penile fracture reported that sexual trauma was the predominant cause of penile fracture, accounting for 69 cases (76.5%). Etiologies were classified as masturbation or penile manipulation, “man‐on‐top”, “doggy style”, “woman‐on‐top”, blunt trauma, and “rolling over” injuries. Among sexual trauma cases, the position at the time of injury varied, with “doggy style” being most frequent (37 cases, 41%), followed by “man‐on‐top” (23 cases, 25.5%) and “woman‐on‐top” (9 cases, 10%) [8].

In contrast, nonsexual causes such as forceful manual manipulation are more prevalent in regions with cultural sensitivities surrounding sexual activity [9]. A study in the United States reported an annual incidence rate of approximately 1.4 cases per 100,000 population [10].

During an erection, the tunica albuginea thins to approximately 0.25–0.5 mm, making it vulnerable to high‐pressure injury [11]. Blunt trauma generates shearing forces that exceed the tunica's tensile strength, leading to rupture. This typically affects one or both corpora cavernosa, and in 1%–38% of cases, the corpus spongiosum and urethra may also be involved [4, 12]. The “pop” sound, followed by rapid detumescence and severe pain, is pathognomonic for this condition.

**FIGURE 6:**
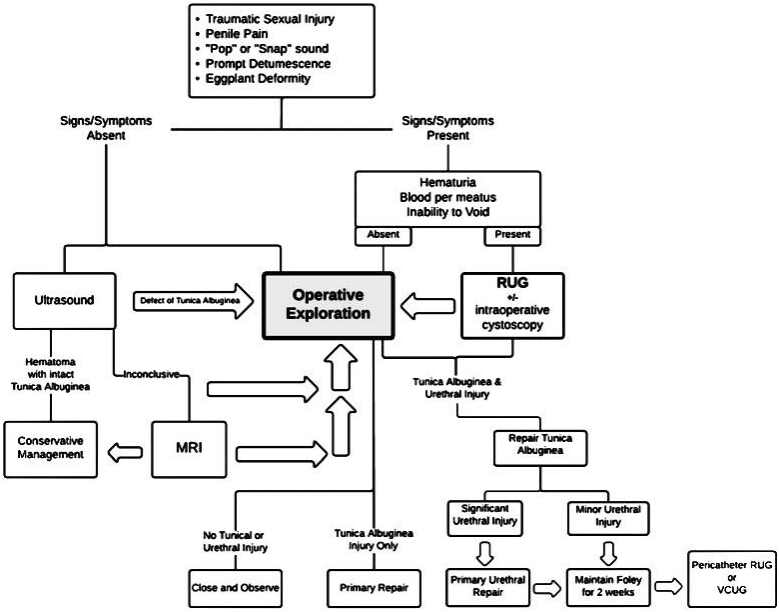
Evaluation and management of suspected penile fracture.

The classic presentation includes penile pain, swelling, deformity, and discoloration, commonly referred to as the “eggplant deformity.” The absence of blood at the urethral meatus or voiding difficulties, as seen in this case, suggests no urethral involvement. In ambiguous cases, ultrasonography can identify tunical defects and hematomas with 71.4% sensitivity and 88.3% specificity, while MRI offers 91.9% sensitivity and 90.6% specificity [13]. However, clinical history and examination are often sufficient for diagnosis in clear‐cut cases. Figure 6 [14]. Diagnosis is primarily clinical [15].

Emergent surgical repair is the gold standard for penile fracture, offering better outcomes than conservative management [5]. Surgical management of penile fracture aims to achieve optimal exposure, hematoma evacuation, precise localization of the defect, repair of the tunica albuginea, secure hemostasis, and when indicated, urethral reconstruction. Incision options include a lateral approach directly over the injury, though this may miss contralateral or corpus spongiosum involvement. A circumferential degloving incision to the penile root, which facilitates complete evaluation of both corpora cavernosa and the corpus spongiosum, and an inguino‐scrotal incision, which provides access to the penile root and dorsal surface [16]. In this case, we used a circumferential degloving incision.

Nonsurgical management, involving bed rest, ice application, and anti‐inflammatory medications, has historically been associated with higher complication rates, including fibrosis and deformity, and is no longer recommended except in extremely mild cases [5].

Most patients achieve favorable outcomes when treated promptly, with 83% regaining normal erectile function [17]. Postoperative complications, including erectile dysfunction and penile curvature, occur in less than 10%–15% of cases when intervention is timely [18]. Sexual abstinence for 6–8 weeks is advised to prevent reinjury and facilitate healing. Long‐term follow‐ups are essential to address late complications, such as Peyronie's disease or psychological distress [19].

## Author Contributions

**Dennis Awedam Achio:** conceptualization, data curation, formal analysis, investigation, methodology, writing – original draft, writing – review and editing. **Richmond Buckner:** writing – review and editing. **Mary Monney‐Bortey:** writing – review and editing. **Celestine Tsogbe:** writing – review and editing. **Bernice Ahiadormey:** writing – review and editing. **Priscilla Mary Tetteh:** writing – review and editing. **Eunice Wilberforce A. Achio:** writing – review and editing.

## Ethics Statement

Ethical approval was not applicable. The study was conducted in accordance with the Declaration of Helsinki.

## Consent

The authors certify that written informed consent was obtained from the patient to publish this report.

## Conflicts of Interest

The authors declare no conflicts of interest.

## Data Availability

The authors have nothing to report.
